# Mass spectrometry-based multimodal approaches for the identification and quantification analysis of microplastics in food matrix

**DOI:** 10.3389/fnut.2023.1163823

**Published:** 2023-04-06

**Authors:** Pengfei Wu, Xiaoyi Wu, Qing Huang, Qinwei Yu, Hangbiao Jin, Minghai Zhu

**Affiliations:** ^1^Department of Basic Research, Nanjing University of Finance and Economics Hongshan College, Nanjing, China; ^2^Co-Innovation Center for Sustainable Forestry in Southern China, Nanjing Forestry University, Nanjing, Jiangsu, China; ^3^College of Materials Science and Technology, Nanjing Forestry University, Nanjing, China; ^4^School of Marine Sciences, Sun Yat-sen University, Guangzhou, China; ^5^Key Laboratory of Microbial Technology for Industrial Pollution Control of Zhejiang Province, College of Environment, Zhejiang University of Technology, Hangzhou, Zhejiang, China

**Keywords:** microplastics, nanoplastics, mass spectrometry, multimodal analysis, food analysis, identification and quantification

## Abstract

**Background:**

Microplastics (MPs) and nanoplastics (NPs) have become emerging contaminants worldwide in food matrices. However, analytical approaches for their determination have yet to be standardized. Therefore, a systematic study is urgently needed to highlight the merits of mass spectrometry (MS) based methods for these applications.

**Purpose:**

The aim of the study is to review the current status of MS-based multimodal analysis for the determination of MPs in food matrices.

**Methods:**

Web of Science and Google Scholar databases were searched and screened until Jan. 2023. Inclusion criteria: “publication years” was set to the last decades, “English” was selected as the “language,” and “research area” was set to environmental chemistry, food analysis and polymer science. The keywords were “microplastics,” “nanoplastics,” “determination,” “identification/quantification,” and “mass spectrometry.”

**Results:**

Traditional spectrometry techniques offer good abilities to conduct the multimodal analysis of MPs in terms of color, shape and other morphologies. However, such technologies have some limitations, in particular the relatively high limits of detection. In contrast, MS-based methods supply excellent supplements. In MS-based methods, gas chromatographic-mass spectrometry (GC-MS), and LC-MS/MS were selected as representative methods for determining MPs in the food matrices, while specialized MS methods (i.e., MALDI-ToF MS and ToF-SIMS) were considered to offer great potential in multimodal analysis of MPs especially when interfaced with the imaging systems.

**Significance:**

This study will contribute to gaining a deeper insight into the assessment of the exposure levels of MPs in human body, and may help build a bridge between the monitoring studies and the toxicology field.

## Introduction

The ever-growing usage of plastics results in increasing the worldwide attention of microplastics (MPs; ≤5 mm) and nanoplastics (NPs; ≤1 mm) pollution and their detrimental consequences on Earth systems (1, 2). Recent data indicate that around 390.7 million tons of plastic were produced in 2021 (3). Considering the outbreak of the COVID-19 pandemic, the extensive usage of personal protective equipment has inevitably exacerbated the upward trend in the growth of plastics usage by about 10.0% annually (4, 5). After use, around 79% of the plastic waste is disposed of improperly under the present management practices (6). Such a large amount of bulk waste further fragments into MPs under the weathering effects, and ∼10% of this ultimately persists in the aquatic environment (6, 7). Recent publications report the widespread presence of MPs in the hydrosphere, atmosphere, and biosphere (8). For example, MPs have been discovered in the polar regions, and the Mariana Trench (9). Through water-vapor exchange, the concentration of MPs in the air can reach as high as 917 items⋅m^–2^⋅d^–1^ (10). The terrestrial environment is also a sink for MPs, and contains about 300–67,500 mg⋅kg^–1^ of MPs (11, 12), especially MPs derived from film mulching (560.0 ± 52.92–2215.56 ± 1549.86 items⋅kg^–1^) (13).

Such an extensive existence of MPs inevitably leads to them being taken up by humans, resulting in a series of detrimental responses, including inflammation, immune impairment and other biochemical consequences (14–16). Cox et al. (17) reported that ingestion is the major pathway for MPs to enter the human body. Herein, the abundance of MPs contained in foods is highlighted. Based on the consumption data for adults and children in the USA, it can be projected that the annual intake of MPs ranges between 81,000 and 123,000 items per year. Another study estimated that the number of MPs could reach 11,000 items per year for one European consumer (18). Our previous study comprehensively estimated the MP exposure to the human body *via* seafood, drinking water, plastic packages and other food items (2). For example, the annual MP exposure by ingestion of mollusks could achieve ∼8.92 × 10^4^ items, while that *via* fish and table salt ranged from 518 to 4600 items⋅year^–1^. These data were one order of magnitude lower than that from mollusks. Such a large discrepancy may result from the variabilities arising from the use of different estimation methods, implying that more accurate determinations for the MP content of food should be established before we can reliably estimate and set the further exposure levels in toxicology studies.

The traditional techniques for MP analysis rely mainly on spectroscopic methods, including Fourier transform infrared spectroscopy (FT-IR) and Raman spectroscopy. Such methods can identify the MPs based on measuring the polymer absorption band according to the specific characteristic functional groups (19). Raman spectrometry is often interfered by the additives on the surface of the MPs, and both of them contain relatively high limits of detection (LOD) at ∼20 μm (20, 21). In combination with an imaging capability, the detection limit theoretically could be decreased to as low as ∼1 μm. However, NPs with smaller sizes (≤1 mm) are often ignored in survey work, resulting in an underestimation of MP abundance in food matrices. For example, Zuccarello et al. (22) found a high abundance of MPs (including NPs, 5.4 × 10^7^ items⋅L^–1^) in the bottled water by scanning electronic microscopy (SEM). However, there was a huge discrepancy with the μ-Raman or the μ-FT-IR results (10–10^3^ items⋅L^–1^) (23, 24). SEM and transmission electron microscopy (TEM) are techniques that may be used to observe MPs; they offer a superior resolution of 1 nm relative to optical microscopy but with a smaller field of view (1 mm^2^). A slightly uneven distribution of MPs in sample plates could also induce huge discrepancies in the results for MP abundance. Additionally, the aforementioned techniques (SEM, TEM) could provide the morphology information of MPs in the food matrices, but such morphological data are not the metrology parameters (*cf* mg⋅kg^–1^, mg⋅L^–1^) that are essential in toxicological research. To ensure compatibility between the monitoring and toxicological field, the application of mass spectrometry (MS) in MP identification and quantitation has begun to attract much attention in recent years.

Mass spectrometry comprises a range of sophisticated analytical techniques that can be applied for molecular detection and determination of material structure and composition. The MS approach has revolutionized analytical practice in chemistry, pharmacy, life science, and inter-related fields (25). With the advantages of rapid and reliable analysis, simple operation, high sensitivity, high-throughput analysis, the MS methods have attracted increasing attention for the direct identification of polymeric structures (16, 26). The basic principle of MS methods relies on the ionization of the MPs followed by separation of molecular constituents according to the *m/z*. The MS analysis further identifies the types and nature of the MPs by determining the repeating unit mass, end groups, and chemical formula (27). The technical challenges in the analytical process rely on the means to ionize the high-molar-mass MP polymers. Therefore, it is vital to appreciate the recent developments in MS as applied to detect the MPs in the food matrices. Herein, we first introduced the current status of using MS to identify MPs in the foods. Moreover, the physiochemical characterization of the size, shape and other characteristics in the food matrices should also be considered. Finally, the challenges and new perspectives for detecting MPs by MS approaches are also outlined and discussed. By exploring these issues, this study will contribute to gaining a deeper insight into the accurate determination of the exposure levels of MPs in the human body, and in so doing may also help build bridges between monitoring studies and toxicological fields.

## Determination of MPs in food matrices by mass spectrometry

**FIGURE 1:**
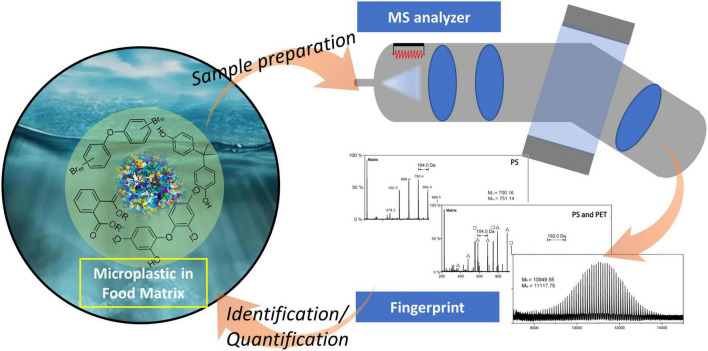
Determination of microplastics by mass spectrometry in food matrix.

Various MS approaches have been developed for the determination of MPs and NPs in food matrices (Figure 1), including gas chromatographic-mass spectrometry (GC-MS), liquid chromatography-tandem mass spectrometry (LC-MS/MS), time-of-flight mass spectrometry (ToF-MS) and other novel MS techniques.

### Gas chromatography-mass spectrometry

Gas chromatography-mass spectrometry is one of the representative methods for determining MPs in the food matrix, especially after coupling with the thermal desorption or pyrolysis devices. In principle, the MPs are first pyrolyzed and fully decomposed into relatively small molecules in an inert environment and then vaporized into the gaseous phase by a micro furnace held at 600–1000°C (28). Finally, the instrument generates the characteristic fingerprint mass spectrum for the sample of interest (29). In addition to polymers, some typical additives contained in the plastics could be analyzed simultaneously.

The GC-MS method has become one of the dominant MS methods for the determination of MPs in foods (Table 1). For example, Peters et al. (30) extracted MPs from benthivore fish and tried to identify the types of plastics by pyrolysis GC-MS. The results showed that a total of 43 MPs were detected, including 30 fibers, 3 fragments, and 10 spheres. Moreover, Polyvinyl chloride (PVC) took the highest priority with 32.6% of the total samples, then followed by polyethylene terephthalate (PET; 9.3%), nylon (9.3%), silicone (2.3%) and epoxy resin (2.3%). Likewise, Liu et al. (31) combined thermal gravimetric analysis (TGA) and GC-MS to identify and quantify MPs in marine mussels. By using the hyphenated approaches, they found up to 1.71 mg of plastic per kg of tissue (mean value: 0.58 mg⋅kg^–1^) in *Mytilus edulis* from six locations along the coast of China, while polyethylene (PE) was found to be the most abundant type of plastics. These two works set the foundation for determining MPs by MS methods in a single species of seafood. Li et al. (32) developed a method to quantify nanoplastic uptake in plants with cucumber by Py-GC/MS. Thereafter, applications were extended by Ribeiro et al. (33), who examined the occurrence and content of MPs in different seafood organisms, i.e., oysters, prawns, squid, crabs, and sardines. The results showed the total concentration of MPs varied highly according to the different species. Meanwhile, Dessì et al. (34) analyzed rice samples by using double-shot pyrolysis gas chromatography/mass spectrometry (Pyr-GC/MS) to estimate the mass concentration of selected plastic polymers in rice. The results showed PE, PET, and polypropylene (PP) were quantifiable in the rice samples (34). The results illustrated that PE was the most frequently detected with 95% in these samples. This is reasonable as they were also widely detected in the air (20). In addition to the MPs, the method also successfully investigated the presence of plastic-related additives and their interaction with the MPs (35). For example, a GC-MS study investigated the relationship between polymethyl methacrylate (PMMA) containing MPs and the environmental contaminant benzo(k)fluoranthene (BkF) where it was found that BkF could be adsorbed by PMMA, thereby decreasing the absorption of contaminants in the fish bodies (31). Uribe-Echeverría et al. (36) utilized GC-MS to investigate the potentially toxic effects of various MPs by determining the plasticizers and additives. It was reported that PVC was the most toxic MPs among polyhydroxybutyrate resin, polylactic acid cups, and polylactic acid/polyhydroxyalkanoate MPs. Additional benefits of GC-MS are that the approach needs minimal sample preparation, and direct injection of the sample can be realized depending upon the matrix. Sample pretreatments in GC-MS involving analyte extraction or separation before injecting the sample but the additional step(s) may be required in the case of complex matrices. However, as with other techniques lacking optical interrogation, GC-MS does not directly provide information on the physical shape of the plastic debris and is destructive, and it should be required high control of experimental conditions (Table 2).

**TABLE 1:** The applications of gas chromatographic-mass spectrometry (GC-MS) and liquid chromatography-tandem mass spectrometry (LC-MS) in microplastics (MPs) determination.

Technique	Samples	Types of MPs	References
Pyr-GC-MS	Raw and treated drinking water	PE > PA > PET > PP > PS*	(35)
Pyr-GC-MS	Spadefish; sand trout; pinfish; kingfish	PVC, PET, nylon	(30)
Pyr-GC-MS	Fish	PE, PP, PET, PS, PVC, PC, PMMA, PA	(49)
TGA-FTIR-GC-MS	Seafoods: mussels	PE, PP, PVC, PS	(31)
TED-GC-MS	Bottled water and other beverages	PET, PE	(50)
LC-MS/MS	Fish fillets	phthalates	(51)
HPLC-MS/MS	Cat and dog foods	PET, PC	(28)
UPLC-MS/MS	Sea turtle	PET, PC	(52)
LC-MS/MS	Fish	PA6, PA66	(40)
LC-MS/MS	Salt	PET, PE	(39)
MALDI-ToF MS	Fish	PET, PS	(26)
ASAP-MS	Bottled water	PHB	(45)
IR-MS	Plastic packaging	PE, PET, PP, PS, PVS, PLA, ABS, PES	(46)
SP-ICP-MS	Food packaging	–	(47)

*PA, polyamide; PE, polyethylene; PET, polyethylene terphtalate; PP, polypropylene; PS, polystyrene; PVC, polyvinyl chloride; PC, polycarbonate; PMMA, polymethyl methacrylate; PHB, poly(hydroxy butyrate).

**TABLE 2:** Advantages and disadvantages of MS technique in MPs analysis.

Technique	Principle	LOD*	Advantages	Disadvantages	References
FT-IR	Polymer absorption band	∼10 μm	Non-destructive; information on the aging degree of MPs	Not suitable for NPs characterization; influenced by the additives; time consuming	(16)
Raman	Polymer absorption band	>1 μm	Non-destructive; Non-contact	Time consuming; heavily influenced by the fluorescent molecules or dyes; time consuming	(16)
SEM	Secondary electron microscopy	>5 nm	Non-destructive; high resolution images	Complex sample preparation; highly depend on sample preparation	(2)
Pyr-GC-MS	Characters fingerprint MS quantification	>1 μg	Minimal sample preparation; highly sensitive	Destruction; complex data processing	(20, 30)
LC-MS/MS	Characters fingerprint MS quantification	LOQ^#^: 6.3 ng/g for PC and 1.5 mg/g for PET;	Highly sensitive; rapid detection speed	Destruction; complex data processing	(28)
MALDI-ToF MS	Characters fingerprint MS quantification	>25 ng	Highly sensitive; rapid detection speed; simple operation; high-throughput analysis	Destruction; complex data processing; solvent requirement	(16, 26)
ToF-SIMS	Characters fingerprint	>20 μm; >70 nm	Highly sensitive; rapid detection speed; simple operation; high-throughput analysis	Destruction; hard to differentiate MPs from samples; complex data processing	(43)

*LOD, limit of detection; #LOQ, limit of quantification.

### Liquid chromatography-tandem mass spectrometry

Liquid chromatography-MS/MS is a workhorse technique widely applied in medicine, environmental and biological analysis (37). In polymer science, many studies have confirmed its applicability to nylon-, PET-, polycarbonate (PC)-based MPs analysis. Similar to GC-MS, the samples typically require extraction, purification and depolymerization before injection into the LC systems (25). In the initial stages of the method, the MPs are extracted and cleaned with solvents, such as NaI and ethanol (38). Thereafter, the small MPs are again decomposed into the monomers, dimers, and oligomers *via* pyrolysis (39). Finally, the small molecules are identified and quantified by tandem MS, hence the molecular mass of the MPs in the matrix may be deduced.

Liquid chromatography-based techniques, offering particularly low detection limits, are widespread in environmental analysis (Table 1). However, these techniques have not been widely utilized for detecting MPs in a food matrix, which might be attributable to the more complex nature of food matrices. The incomplete degradation of substances that form during pyrolysis could contaminate the ionization source, resulting in damage to the device. To date, only a few studies have adopted the method for determining MPs in food samples. Wang et al. (40) studied the abundance of nylon 6 in fish by determining the content of 6-aminocaproic acid and adipic acid, which were in the order of 10.8 and 39.6 mg⋅kg^–1^, respectively, while that of nylon 66 ranged between 13.4 and 49.2 mg⋅kg^–1^. These contents are considered to be relatively high for fish when considering concentrations in other matrixes, e.g., sludge (nylon 6: 15.2 mg⋅kg^–1^; nylon 66: 48.2 mg⋅kg^–1^), sediments (nylon 6: 0.725–2.54 mg⋅kg^–1^; nylon 66: 8.15–15.4 mg⋅kg^–1^). In bivalves, the same group reported the presence of PC- and PET-based MPs by determining bisphenol A (BPA) and *p*-phthalic acid (PTA), respectively (39). After careful calculation, the PC MPs in bivalve ranged from 2.33 to 4.41 mg/kg, while the concentrations for PET MPs were calculated to be 75.4–17.5 mg/kg, respectively. In addition to seafood, the group also determined the concentrations of PC and PET MPs in salt samples, which were calculated to be 0.088 and 0.102 mg/kg, respectively (39). Not only in human foods, but the PC and PET MPs were also found in pet food (cat and dog) in the United States (28). Also by determining the concentrations of BPA and PTA, it was discovered that there were about 0.23 and 61 mg/kg of PC and PET MPs in cat food, and 0.16 and 30 mg/kg in dog food, respectively. However, it remains a challenge to directly compare the exposure levels to MPs in foods intended for human and pet consumption. This technique is destructive in nature. The detection strategy also does not provide information on the count, size, color and shape of MPs and NPs, but rather it yields information on the mass and quantity of monomers released during depolymerization (Table 2) (34).

### Time-of-flight mass spectrometry

Time-of-flight mass spectrometry is a MS technique whereby an ion’s *m/z* is determined *via* the time each ion takes to reach the detector. Under the same electrical field with known strength, ions of the same charge are accelerated with the same kinetic energy. Due to the discrepancy of the mass, each ion would have a different corresponding velocity. In other words, it means that the time each ion takes to reach the detector would vary and be inversely proportional to the molecular mass of the ion. On this basis, the *m/z* of the ions would provide a means for identification. In this MS approach, two prevalent techniques have found application in MPs analysis, namely, matrix assisted laser desorption/ionization-time of flight mass spectrometry (MALDI-ToF MS) and time of flight secondary ionization mass spectrometry (ToF-SIMS).

Matrix assisted laser desorption/ionization-time of flight mass spectrometry is a powerful MS technique often applied for analysis of high-molecular weight substances. The device consists of three essential modules, MALDI as the ion source, ToF as the mass separation, and the MS detector for the mass analysis (16). Once the MPs are ionized gently the ions are conveyed *via* a matrix vaporized by the laser energy, the [polymer]^+^ or [polymer]metal^+^ ions would then be separated by their *m/z* in the ToF system. The technique has been used successfully to determine different types of MPs (41). Recently, PS and PET MPs have been identified and quantified in complex food matrixes (26). In the latter study, a thermal pretreatment (380°C) was used to facilitate the fragmentation of macromolecules thereby enhancing the intensities of the characteristic peaks. A detection limit of 25 ng for PS MPs was obtained with a good linear relationship (*R*^2^ 0.986) for signal quantification. The method was used to determine PS MPs in fish samples where the concentrations of PS MPs in fish ranging between 0.068 and 0.146 mg⋅g^–1^. These results demonstrated the reliability and effectiveness of the proposed technique for determining PS MPs in food samples. When combined with imaging system, the multimodal analysis of MPs was realized, and additional information including shape, size, color, degree of aging, and chemical composition (42) were also obtained. This approach may be considered as one of the preferred analytical techniques in future MPs analysis.

Time of flight secondary ionization mass spectrometry is based on detecting secondary ions, electrons or even neutrals which are generated as a result of the primary ions interacting with the sample surface. Once the primary ion gun is aligned with the sample surface which is then interrogated, a ToF-SIMS mass spectrum is generated by summing the detected secondary ion intensities and plotting them against the mass channels. The technique is suitable for the analysis of inorganic and organic substances. Similar to the MALDI-ToF MS, it is also possible to conduct rapid MS scanning and present the organic ion graphs when coupled with an imaging system. Hence, in theory it is possible to supply information on MP sizes and their distributions in the food matrix. Recently, Jungnickel et al. (43) reported that PE MPs could be determined by ToF-SIMS, the main ions detected being [C_*n*_H_2*n*–1_]^+^, [C_*n*_H_2*n*+1_]^+^, and [C_3_H_3_(CH_2_)_*n*_]^+^ in sand samples. Furthermore, another study was conducted on four types of MPs (e.g., PVC, PET, nylon 6, and PP in soil samples. However, these studies focused mainly on environmental analysis (44).

### Other novel MS techniques

Even though the above multimodal MS methods have been largely used for MP analysis, there are also some other MS-based techniques, including the atmospheric solids analysis probe mass spectrometry (ASAP-MS) (45), isotope ratio mass spectrometry (IR-MS) (46), and single particle-inductively coupled plasma mass spectrometry (SP-ICP-MS) which are impacting the field (47). These MS methods exhibit great potential for MPs analysis in the food matrix although they are still considered to be at a developmental stage. Vitali et al. (45) employed ASAP-MS for the chemical analysis of PS, PET, PC, PE, and other common plastics, while undertaking image analysis for characterization of the MPs. In real samples, good qualitative and quantitative results, such as the MP number, size and shape distribution, were demonstrated. Thus, in this way, characterization of MPs may proceed allowing full information related to contamination to be collected. For the SP-ICP-MS technique, Birch et al. (47) undertook a systematic study on characterization of common MPs by using the carbon isotopic analysis (^13^C/^12^C) techniques. Full compatibility with spectroscopic techniques and IR-MS image analysis approaches was undertaken to compare the advantages/disadvantages of the present technologies in measuring the MP content of bottled water (Table 2) (46). In a similar vein, in SP-ICP-MS, the ^13^C isotope can be exploited to screen the MPs in personal care products thus enabling studies on how MPs released from food packaging, which is also a crucial way for MPs entering the food matrix.

## Challenges and future perspectives

Microplastics in the last few decades have become a crucial topic in the environmental and health field. Lots of attention has focused on the MP determination, exposure routes, and health effects, and clearly analytical techniques provide the foundation for studying the health risks. Most of the modern analytical techniques have proved to be suitable for the analysis of micron sized and submicron sized plastics, while the identification and quantification of NPs are hindered at present (Challenge 1). Another significant challenge is that the mass of plastics is relatively low thus it is not possible to weigh accurately MPs in samples using gravimetric methods (e.g., balance) are difficult to weigh them reliably (Challenge 2). A further complication relates to the MPs with other substances could complicate the subsequent analysis (Challenge 3). Therefore, development of reliable and stable techniques are a high priority for studying the health effects of MPs in foods. In devising mitigation approaches, MS-based analytical strategies exhibited a high potential for MPs analysis in the food matrix. First and most important, these techniques can fill the knowledge gap within the field regarding the abundance of MPs in the food matrix and, moreover, facilitate on their potential interactions with cells or living organisms after ingestion (48). Second, the MS-based multimodal techniques in conjunction with imaging systems could supply information of MPs, such as the size, shape, and even aging (16). Third, the chemical additives and other relevant contaminants could be analyzed during the determination of the MPs.

## Conclusion

This review has summarized the principles and applications of MS methods for the determination of MPs in the food matrix. Among the MS techniques, GC-MS has been used extensively used in the analysis of the polymer compositions, plasticizers, and other additives in MPs. In addition, previous studies have found that LC-MS of microplastics are used less frequently. Notably, the novel multimodal MS methods including MALDI-ToF MS, ToF-SIMS, ASAP-MS, IR-MS, and SP-ICP-MS, could enhance the characterization of MPs in many circumstances. Moreover, confronting the challenges and future perspectives have also been highlighted in terms of what is currently known, especially the relationship regarding the abundance of MPs in the food matrix and potential interactions with cells or living organisms after ingestion. Therefore, this review could be of great importance given the increasing number of MPs that are being detected in the environment and food matrices. Further, the review could also provide a deeper insight into accurately measuring the exposure levels of MPs in the human body *via* ingestion, which could help to fill the knowledge gap regarding the abundance of MPs and their toxicity.

## Author contributions

PW and XW wrote the original draft of the manuscript. PW, QH, and QY conducted the searching processes. HJ reviewed and edited the manuscript. MZ supervised the manuscript and did the funding acquisition. All authors contributed to the article and approved the submitted version.
